# Central obesity is important but not essential component of the metabolic syndrome for predicting diabetes mellitus in a hypertensive family-based cohort. Results from the Stanford Asia-pacific program for hypertension and insulin resistance (SAPPHIRe) Taiwan follow-up study

**DOI:** 10.1186/1475-2840-11-43

**Published:** 2012-04-26

**Authors:** I-Te Lee, Yen-Feng Chiu, Chii-Min Hwu, Chih-Tsueng He, Fu-Tien Chiang, Yu-Chun Lin, Themistocles Assimes, J David Curb, Wayne H-H Sheu

**Affiliations:** 1 Division of Endocrinology and Metabolism, Department of Internal Medicine, Taichung Veterans General Hospital, Taichung, 407, Taiwan; 2 Institute of Medicine, Chung Shan Medical University, Taichung, 402, Taiwan; 3 School of Medicine, National Yang-Ming University, Taipei, 112, Taiwan; 4 Division of Biostatistics and Bioinformatics, Institute of Population Health Sciences, National Health Research Institutes, Miaoli, 350, Taiwan; 5 Division of Endocrinology and Metabolism, Department of Internal Medicine, Taipei Veterans General Hospital, Taipei, 112, Taiwan; 6 Division of Endocrinology and Metabolism, Department of Internal Medicine, Tri-Service General Hospital, National Defense Medical Center, Taipei, 114, Taiwan; 7 Department of Internal Medicine, National Taiwan University Hospital, Taipei, 100, Taiwan; 8 Department of Medicine, Stanford University School of Medicine, Stanford, CA, 94305, USA; 9 John A. Burns School of Medicine, University of Hawaii, Honolulu, HI, 96813, USA

**Keywords:** Metabolic syndrome, Incidence, New-onset diabetes, Obesity

## Abstract

**Background:**

Metabolic abnormalities have a cumulative effect on development of diabetes, but only central obesity has been defined as the essential criterion of metabolic syndrome (MetS) by the International Diabetes Federation. We hypothesized that central obesity contributes to a higher risk of new-onset diabetes than other metabolic abnormalities in the hypertensive families.

**Methods:**

Non-diabetic Chinese were enrolled and MetS components were assessed to establish baseline data in a hypertensive family-based cohort study. Based on medical records and glucose tolerance test (OGTT), the cumulative incidence of diabetes was analyzed in this five-year study by Cox regression models. Contribution of central obesity to development of new-onset diabetes was assessed in subjects with the same number of positive MetS components.

**Results:**

Among the total of 595 subjects who completed the assessment, 125 (21.0%) developed diabetes. Incidence of diabetes increased in direct proportion to the number of positive MetS components (P ≪ 0.001). Although subjects with central obesity had a higher incidence of diabetes than those without (55.7 vs. 30.0 events/1000 person-years, P ≪ 0.001), the difference became non-significant after adjusting of the number of positive MetS components (hazard ratio = 0.72, 95%CI: 0.45-1.13). Furthermore, in all participants with three positive MetS components, there was no difference in the incidence of diabetes between subjects with and without central obesity (hazard ratio = 1.04, 95%CI: 0.50-2.16).

**Conclusion:**

In Chinese hypertensive families, the incidence of diabetes in subjects without central obesity was similar to that in subjects with central obesity when they also had the same number of positive MetS components. We suggest that central obesity is very important, but not the essential component of the metabolic syndrome for predicting of new-onset diabetes. (Trial registration: NCT00260910, ClinicalTrials.gov).

## Background

It is well known that insulin resistance is associated with hypertension [1]. Within hypertensive families, interestingly, subjects without hypertension also have high insulin resistance, which is similar in degree to that of subjects with present hypertension [2]. On the other hand, obesity imposes an additional impact on insulin resistance in the members of a hypertensive family, regardless of whether they have hypertension or not [3,4]. Although high insulin resistance has been observed among hypertensive family members, it was reported that the prevalence of diabetes was not significantly higher in comparison to those without a family history of hypertension [5].

Metabolic syndrome (MetS) is composed of a cluster of cardiovascular risks [6]. A higher number of positive MetS components is usually correlated with a higher risk of developing cardiovascular disease and diabetes [7]. Central obesity is considered a pivotal component in MetS, and it is defined as the only indispensable component of MetS based on the diagnostic criteria of the International Diabetes Federation (IDF), [8-10]. It is notable that subjects with central obesity often have more metabolic disorders and thus are at greater risk of developing diabetes than those without central obesity [11]. Even in subjects without obesity, it has been reported that a higher body mass index (BMI) tends to correlate with a higher number of positive MetS components [12,13]. However, it is debatable whether central obesity is still indispensable among subjects with the same number of positive MetS components, and whether subjects without central obesity should not be diagnosed as having MetS [9,14-16]. Therefore, in this study, we wanted to test the hypothesis that central obesity would be associated with more cases of new-onset diabetes in subjects within hypertensive families characterized by high insulin resistance.

## Methods

This cohort study was a five-year epidemiological follow-up study conducted in Taiwan, which was an extension of the previously published Stanford Asia-Pacific Program for Hypertension and Insulin Resistance (SAPPHIRe) [17]. Briefly, the study was family-based and enrolled concordant sib pairs (both hypertensive sibs) and discordant sibs (one with hypertension and one without hypertension). The study subjects were aged between 35 and 60 years, but those who were pregnant, or had chronic kidney disease (serum creatinine ≫ 133 μmol/L), cancer or other severe chronic diseases such as liver cirrhosis were excluded from the study [18]. This follow-up study was only conducted at four teaching hospitals in Taiwan. All subjects were Chinese and were excluded if they had diabetes at baseline. The study was approved by the Institutional Review Boards at all participating sites. Written informed consent was obtained from all subjects (Trial registration: NCT00260910, ClinicalTrials.gov).

The anthropometric assessments were performed in the morning after an overnight fast. The measurements of body weight (DETECTO, Cardinal Scale Manufacturing Co., Webb City, MO, USA) and body height (Pharmacia Taiwan Inc., Taipei, Taiwan) were performed after participants removed their shoes or any heavy clothing. Waist circumference (kp-1508, King Life, Taipei, Taiwan) was measured at the level of the umbilicus after expiration while the participant breathed quietly and regularly. Blood pressure was detected using the DINAMAP^TM^ Vital Signs Monitor (Model 1846 SX/P) after subjects had been sitting at rest for 10 min. The average of the second and the third readings from three separate readings with intervals of 1 min was recorded. Overnight fasting blood samples were collected for measurements of glucose, insulin and lipoprotein profiles after obtaining the anthropometric measurements. The homeostasis model assessment for insulin resistance (HOMA-IR) index was calculated using the formula of [fasting insulin (μU/mL) * fasting glucose (mmol/L)]/22.5 [17]. A standard 75 g oral glucose tolerance test (OGTT) was performed for measurement of post-challenge plasma glucose level at 120 minutes (2 hr pc glucose) at baseline and at the end of 5-year follow-up, except when diabetes had been diagnosed in clinical practice. The medical records about diabetes were traced during the study.

Based on the Third Report of the National Cholesterol Education Program (NCEP), MetS is defined as the presence of three or more of the following components [6]: (1) waist circumference ≥ 90 cm in men or ≥ 80 cm in women [10,19], (2) triglycerides ≥ 150 mg/dl (1.7 mmol/L), (3) High-density lipoprotein (HDL) cholesterol ≪ 40 mg/dl (1.0 mmol/L) in men or ≪ 50 mg/dl (1.3 mmol/L) in women, (4) blood pressure ≥ 130/85 mmHg or using antihypertensive medications and (5) fasting glucose ≥ 100 mg/dl (5.6 mmol/L) [10]. The diagnosis of diabetes in the current study was based on the presence of one or more of the following criteria: (1) fasting glucose ≥ 126 mg/dl (7.0 mmol/L), (2) OGTT ≥ 200 mg/dl (11.1 mmol/L), or (3) use of anti-diabetic agents.

## Statistical analyses

All descriptive data were presented as mean ± standard deviation or number with percentage of subjects. All subjects were classified as having central obesity if waist circumference ≥ 90 cm in men or ≥ 80 cm in women. Regression analyses based on the generalized estimating equation (GEE) approach were conducted to assess the significance of the differences among groups. The GEE approach was applied to account for corrections among subjects within a family. Cox proportional hazard models, which accounted for within-family correlations, were used to analyze the associations of each MetS component with incidence of diabetes. All the statistical analyses were performed using SAS software version 9.1.3 (SAS, Cary, NC, USA).

## Results

**Figure 1:**
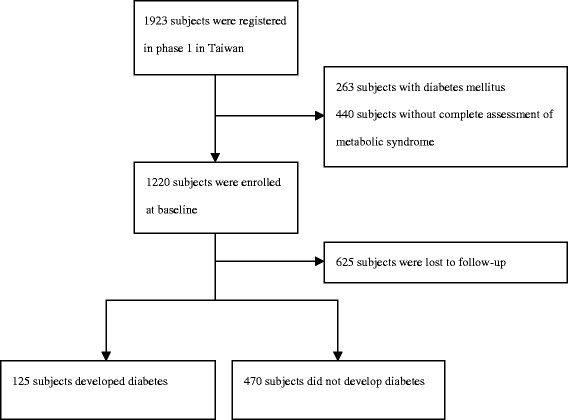
Flow diagram of enrollment of study subjects.

**Figure 2:**
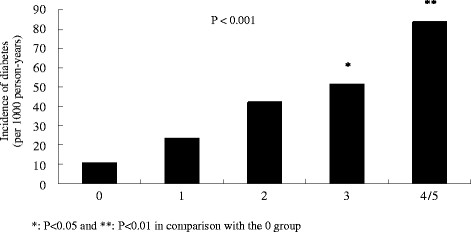
Incidence of diabetes among different numbers of positive components in metabolic syndrome.

Of the total number of 1220 study subjects enrolled, 625 subjects did not complete the assessments in the follow-up phase. Data from the 595 subjects with complete information about MetS and diabetes were collected and analyzed (Figure 1). There were no significant differences in baseline characteristics, including gender, age, smoking status, BMI, blood pressure, glucose, lipid profiles and HOMA-IR, between the subjects who were lost during follow-up and those whose complete data were collected. All study subjects were classified into one of five groups based on the number of positive MetS components: 0, 1, 2, 3, and 4/5 factors. The characteristics of these five groups are shown in Table 1. At the end of the study, the number of subjects diagnosed with diabetes were 4 (5.2%), 19 (12.0%), 34 (21.9%), 36 (27.1%) and 32 (44.4%) in groups 1 to 5, respectively. As expected, we found a higher number of positive MetS components correlated with a higher incidence of diabetes (Figure 2).

**Table 1:** The baseline characteristics of study subjects categorized by number of positive components in metabolic syndrome

	0	1	2	3	4/5	P^#^
No. of patients	77	158	155	133	72	
Male gender, n (%)	28 (36.4%)	64 (40.5%)	69 (44.5%)	68 (51.1%)*	45 (62.5%)***	<0.001
Age (years)	45.6±7.5	47.2±8.4	47.8±8.8	49.9±9.1**	50.3±8.8**	<0.001
Smoking (packs/day*year)	2.8±6.42	3.1±8.1	4.7±10.7	5.3±10.1*	9.4±13.5***	<0.001
Body Mass Index (kg/m^2^)	21.9±1.9	23.7±2.4***	25.2±2.9***	26.6±2.9***	27.7±2.4***	<0.001
Waist circumference (cm)	74.7±6.1	78.5±7.8***	83.1±9.7***	88.6±9.2***	92.0±6.8***	<0.001
Systolic BP (mmHg)	103±10	121±26***	132±24***	137±23***	144±23***	<0.001
Diastolic BP (mmHg)	64±7	74±14***	78±14***	81±13***	84±12***	<0.001
Fasting glucose (mmol/L)	4.7±0.4	4.8±0.4	4.9±0.4*	5.0±0.6***	5.4±0.6***	<0.001
2hr pc glucose (mmol/L)	6.5±1.8	6.9±1.5	7.0±1.9*	7.5±1.7***	8.4±1.8***	<0.001
Insulin (μU/mL)	4.2±1.9	5.7±3.1***	7.3±4.6***	8.5±4.6***	11.5±5.7***	<0.001
HOMA-IR	0.9±0.4	1.3±0.7***	1.6±1.1***	1.9±1.1***	2.8±1.5***	<0.001
Triglyceride (mmol/L)	0.8±0.3	1.0±0.4***	1.3±0.6***	1.6±0.9***	2.4±1.1***	<0.001
HDL cholesterol (mmol/L)	1.5±0.3	1.2±0.3***	1.1±0.3***	1.0±0.2***	0.9±0.2***	<0.001
Total cholesterol (mmol/L)	4.6±0.9	4.8±1.1	4.8±0.9	4.8±1.1	4.9±0.9	0.177
LDL cholesterol (mmol/L)	2.7±0.8	3.1±1.1	3.1±0.9	3.1±1.0	3.0±0.9	0.393

#: P value among the five group.

*: P<0.05, **: P<0.01 and ***: P<0.001 in comparison with the 0 group.

Abbreviations: 2hr pc glucose= postchallenge plasma glucose level at 120 min, BP= blood pressure, HDL= high-density lipoprotein, HOMA-IR= homeostasis model assessment for insulin resistance and LDL= low-density lipoprotein.

**Figure 3:**
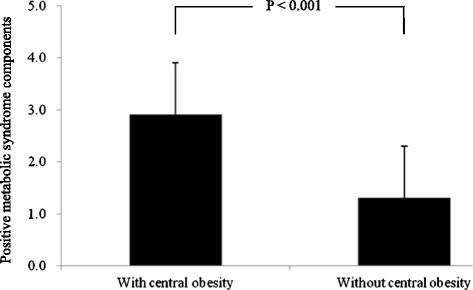
The numbers of positive components in metabolic syndrome between subjects with central obesity and without central obesity.

In order to assess the effect of central obesity on development of diabetes, all study subjects were classified into with central obesity group or without central obesity group according to the criterion of waist circumference. Subjects with central obesity had higher blood pressure, higher triglycerides, lower HDL cholesterol and higher glucose levels (all P values less than 0.001). A higher incidence of diabetes was also noted in the with central obesity group than in the without central obesity group (55.7 vs. 30.0 cases/1000 person-years, P = 0.009) (Table 2). Since obese subjects had a higher number of positive MetS components (2.9 ± 1.0) than those without obesity (1.3 ± 1.0) (Figure 3), the hazard ratio was calculated after adjusting for the number of positive MetS components, age, gender and smoking. We found that there was no significant difference in development of diabetes between subjects with central obesity and those without central obesity (hazard ratio = 0.72, 95%CI = 0.45-1.13, P = 0.151).

**Table 2:** The baseline characteristics of study subjects categorized by central obesity

	With central obesity	Without central obesity	P
No. of patients	234	361	
Male gender, n (%)	105 (44.9%)	169 (46.8%)	0.755
Age (years)	50.0±9.4	46.9±8.0	<0.001
Smoking (packs/day)	6.4±11.8	3.6±8.5	<0.001
Waist circumference (cm)	91.9±7.3	77.4±7.0	<0.001
Systolic BP (mmHg)	135±25	123±25	<0.001
Diastolic BP (mmHg)	79±14	75±14	<0.001
Fasting glucose (mmol/L)	5.1±0.6	4.9±0.5	<0.001
Triglyceride (mmol/L)	1.5±0.8	1.3±0.8	<0.001
HDL cholesterol (mmol/L)	1.1±0.3	1.2±0.3	<0.001
HOMA-IR	2.1±1.3	1.3±0.9	<0.001
Incidence of diabetes (per 1000person-years)	55.7	30.0	0.009

Abbreviations: BP= blood pressure, HDL= high-density lipoprotein, HOMA-IR= homeostasis model assessment for insulin resistance.

When analyses were limited to subjects with three positive MetS components, there was no significant difference in baseline HOMA-IR between subjects with and those without central obesity (Table 3). The incidence of diabetes in subjects with central obesity was not significantly different from the incidence in those without central obesity (55.3 vs. 43.9 cases/1000 person-years, P = 0.792). After adjusting for age, gender and smoking, there was still no significant difference in incidence of diabetes between subjects with and those without central obesity (hazard ratio = 1.04, 95% CI = 0.50 - 2.16, P = 0.918).

**Table 3:** The baseline characteristics of study subjects with three positive components of metabolic syndrome

	With central obesity	Without central obesity	P
No. of patients	89	44	
Male gender, n (%)	42 (47.2%)	26 (59.1%)	0.163
Age (years)	50.6±9.6	48.4±7.7	0.004
Smoking (packs/day)	5.5±10.3	4.9±9.6	0.616
Body Mass Index (kg/m^2^)	27.4±2.8	24.8±2.1	<0.001
Waist circumference (cm)	92.3±8.2	81.2±6.2	<0.001
Systolic BP (mmHg)	138±23	136±22	0.500
Diastolic BP (mmHg)	81±14	81±12	0.889
Fasting glucose (mmol/L)	5.0±0.6	5.1±0.6	0.200
2hr pc glucose (mmol/L)	7.3±1.8	7.9±1.4	0.112
Triglyceride (mmol/L)	1.4±0.8	2.1±0.7	<0.001
HDL cholesterol (mmol/L)	1.1±0.2	0.9±0.2	<0.001
Total cholesterol (mmol/L)	4.8±1.0	4.7±1.2	0.772
LDL cholesterol (mmol/L)	3.2±0.9	3.0±1.1	0.564
HOMA-IR	1.9±1.1	1.9±1.0	0.989
Incidence of diabetes (per 1000person-years)	55.3	43.9	0.792

Abbreviations: 2hr pc glucose= post-challenge plasma glucose level at 120 minutes, BP= blood pressure, HDL= high-density lipoprotein, HOMA-IR= homeostasis model assessment for insulin resistance and LDL= low-density lipoprotein.

Among subjects without central obesity, none of the individual MetS components showed particular relevance to subsequent risk of new-onset diabetes. In contrast, hypertriglyceridemia and impaired fasting glucose were the most important predictors for diabetes in the subjects with central obesity (Table 4).

**Table 4:** Cox regression models for the effects of metabolic syndrome on the incidence of diabetes in study subjects with central obesity or without central obesity

	Incidence of diabetes					
	Without central obesity (n=361)			With central obesity (n=234)		
	HR	95% CI	P	HR	95% CI	P
Hypertension	1.61	(0.86,3.00)	0.137	1.46	(0.71,3.00)	0.309
Low HDL cholesterol	1.69	(0.92,3.09)	0.090	1.11	(0.63,1.98)	0.714
Hypertriglyceridemia	1.17	(0.56,2.44)	0.682	2.34	(1.44,3.80)	0.001
Impaired fasting glucose	1.83	(0.77,4.33)	0.168	3.00	(1.63,5.54)	<0.001

Adjusted for age, gender, smoking and waist circumference.

Abbreviations: CI = confidence interval, HDL= high-density lipoprotein, HR = hazard ratio.

## Discussion

In a hypertensive family cohort characterized by insulin resistance [2-4], we found a cumulative effect of MetS components on the risk of developing diabetes, which was relatively similar to that reported in the West of Scotland Coronary Prevention Study (WOSCOPS) [7]. In a high insulin-resistance population, the effect of MetS accumulation was also observed in subjects with impaired fasting glucose [20]. Our most important finding is that the risk of developing new-onset diabetes was associated with the number of positive MetS components, but not with central obesity. When analyses were limited to those subjects with three MetS components, neither insulin resistance (expressed by HOMA-IR) at baseline, nor diabetes incidence during follow-up was significantly different between subjects with and without central obesity. Previous reports that subjects with central obesity had a higher risk of developing diabetes might be based on the evidence that these individuals has a higher number of associated MetS components [21,22]. In accord with our observations, results from the Anglo-Scandinavian Cardiac Outcomes Trial (ASCOT) demonstrated that MetS was a better predictor of development of diabetes than BMI in hypertensive subjects [23]. Nevertheless, Yasuda T, et al. [24] found that central obesity did not impose any additional influence on carotid atherosclerosis among subjects with the same number of positive MetS components in a cross-sectional study. Non-obese subjects with metabolic syndrome also had a similar risk of developing cardiovascular disease compared with obese individuals in prospective observations [25,26]. In fact, a joint interim statement from several associated organizations has suggested that MetS criteria need to be unified and waist circumference might not be the prerequisite for the criteria [27].

The proportion of new-onset diabetes among the members of hypertensive families in the present study was higher than that in the WOSCOPS and also than that reported in a previous Chinese community survey [7,28]. On the other hand, Shirakawa et al. [5] had reported that a family history of hypertension was associated with the presence of hypertension but not diabetes in a previous cross-sectional study. According to the high cumulative incidence of diabetes in our prospective cohort, however, a family history of hypertension does provide important information in predicting development of diabetes. These findings might be due to the hyperinsulinemia observed in hypertensive family [2]. High fasting insulin is associated with many metabolic abnormities and can lead MetS to progress [29]. Similarly, in the present study, insulin resistance with high fasting insulin levels had a positive association with the number of MetS components and incidence of diabetes. However, we did not further assess the possible mechanisms connecting a family history of hypertension and the incidence of diabetes. It has been reported that circulating aldosterone might associate with insulin resistance [30], and the autonomic imbalance is probably related to diabetes [31].

The components of MetS partially share the same mechanisms, and frequently coexist. Although the predictive power of MetS seems not as strong as the sum of all its components, the diagnosis of MetS is important as a superimposed risk on traditional risk factors. [20,32,33]. On the other hand, subjects without obesity probably have several metabolic abnormalities which can lead to an increased risk of diabetes [34,35]. In this regard, we should not ignore the potential risk in subjects without central obesity. Furthermore, several studies showed that impaired fasting glucose is a strong predictor of development of diabetes [20,23,28]. In addition to glucose, fasting triglyceride was the other independent predictor of onset of diabetes in Chinese subjects [28]. In particular, these finding is obvious in the subjects with central obesity in our data (see Table 4). In fact, lipotoxicity by deposition of triglycerides and free fatty acids may be associated with insulin resistance and beta-cell dysfunction [36,37]. Therefore, it is important to closely monitor or treat subjects with high levels of triglycerides and glucose because these two components are clearly associated with development of diabetes, especially in those with central obesity [37,38].

There were a few limitations in the present study. First, central obesity is one of the present MetS criteria, so subjects with central obesity wound inevitably have a higher number of positive MetS components than those without central obesity if we did not select subjects with the same number of metabolic abnormalities. We focused on subjects with only three positive MetS components in the present study. No analyses of data for subjects with four positive components, the maximal number of abnormalities in the without central obesity group, were performed because there were fewer cases in this subgroup. Secondary, as the aim of the present study was to investigate MetS, we used the criterion of waist circumference to define central obesity. However, BMI is often assessed in the study of metabolically obese, normal-weight (MONW) subjects [35]. In addition, we did not find significant differences in predicting diabetes in subjects with normal waist circumference. However, some adipocytokines, including serum leptin, are associated with MetS and insulin resistance in non-obese as well as obese subjects [39-41]. Further studies on predisposing factors leading to diabetes are needed.

## Conclusions

In Chinese hypertensive families, the incidence of diabetes in subjects without central obesity was similar to that in subjects with central obesity when they also had the same number of positive MetS components. We suggest that central obesity is very important, but not the essential component of the metabolic syndrome for predicting of new-onset diabetes.

## Abbreviations

BMI = Body mass index; GEE = Generalized estimating equation; HOMA-IR = Homeostasis model assessment for insulin resistance; IDF = International Diabetes Federation; MetS = Metabolic syndrome; MONW = Metabolically obese normal-weight; NCEP = National Cholesterol Education Program; OGTT = Oral glucose tolerance test; PC = Post-challenge; SAPPHIRe = Stanford asia-pacific program for hypertension and insulin resistance.

## Competing interests

The authors declare that they have no competing interests.

## Authors’ contribution

ITL wrote the manuscript, contributed to discussions and researched data. YFC wrote the manuscript, contributed to discussions and researched data. CMH contributed to discussions and researched data. CTH researched data. FTC researched data. YCL researched data. TA researched data. DC researched data. WHHS reviewed/edited the manuscript, contributed to discussions and researched data. All authors read and approved the final manuscript.
